# Saffron (*Crocus sativus* L.): A Source of Nutrients for Health and for the Treatment of Neuropsychiatric and Age-Related Diseases

**DOI:** 10.3390/nu14030597

**Published:** 2022-01-29

**Authors:** Adil El Midaoui, Imen Ghzaiel, Dominique Vervandier-Fasseur, Mohamed Ksila, Amira Zarrouk, Thomas Nury, Farid Khallouki, Aboubaker El Hessni, Salama Ouazzani Ibrahimi, Norbert Latruffe, Réjean Couture, Omar Kharoubi, Fatiha Brahmi, Sonia Hammami, Olfa Masmoudi-Kouki, Mohamed Hammami, Taoufik Ghrairi, Anne Vejux, Gérard Lizard

**Affiliations:** 1 Department of Pharmacology and Physiology, Faculty of Medicine, University of Montreal, Montreal, QC H3C 3J7, Canada; rejean.couture@umontreal.ca; 2 Department of Biology, Faculty of Sciences and Techniques Errachidia, Moulay Ismail University of Meknes, Errachidia 52000, Morocco; farid_khallouki@yahoo.fr; 3 Laboratory of Genetics, Neuroendocrinology, and Biotechnology, Department of Biology, Faculty of Sciences, Ibn Tofail University, Kenitra 14020, Morocco; elhessni70@yahoo.fr (A.E.H.); salama.ouazzani.ibrahimi.1@gmail.com (S.O.I.); 4 Team ‘Biochemistry of the Peroxisome, Inflammation and Lipid Metabolism’, University of Bourgogne Franche-Comte, 21000 Dijon, France; imenghzaiel93@gmail.com (I.G.); mohamedksila44@gmail.com (M.K.); thomas.nury@u-bourgogne.fr (T.N.); norbert.latruffe@u-bourgogne.fr (N.L.); anne.vejux@u-bourgogne.fr (A.V.); 5 Lab-NAFS ‘Nutritio—Functional Food & Vascular Health’, Faculty of Medicine, LR12ES05, University Monastir, Monastir 5000, Tunisia; zarroukamira@gmail.com (A.Z.); sonia.hammami@fmm.rnu.tn (S.H.); mohamed.hammami@fmm.rnu.tn (M.H.); 6 Team OCS, Institute of Molecular Chemistry (ICMUB UMR CNRS 6302), University of Bourgogne Franche-Comte, 21000 Dijon, France; dominique.fasseur@u-bourgogne.fr; 7 Laboratory Neurophysiology, Cellular Physiopathology and Valorisation of Biomolecules, (LR18ES03), Department of Biology, Faculty of Sciences, University Tunis El Manar, Tunis 2092, Tunisia; olfa.masmoudi@fst.utm.tn (O.M.-K.); taoufik.ghrairi@fst.utm.tn (T.G.); 8 Laboratory of Biochemistry, Faculty of Medicine, University of Sousse, Sousse 4000, Tunisia; 9 Laboratory of Experimental Biotoxicology, Biodepollution and Phytoremediation, Faculty of Life and Natural Sciences, University Oran1 ABB, Oran 31000, Algeria; omarkharoubi@gmail.com; 10 Laboratory Biomathématique, Biochimie, Biophysique et Scientométrie, Faculté des Sciences de la Nature et de la Vie, Université de Bejaia, Bejaia 06000, Algeria; fatiha.brahmi@univ-bejaia.dz

**Keywords:** saffron, crocus sativus, crocins, crocetin, picrocrocin, safranal, nutrients, neuropsychiatric diseases, age-related diseases

## Abstract

Saffron (*Crocus sativus* L.) is a medicinal plant, originally cultivated in the East and Middle East, and later in some Mediterranean countries. Saffron is obtained from the stigmas of the plant. Currently, the use of saffron is undergoing a revival. The medicinal virtues of saffron, its culinary use and its high added value have led to the clarification of its phytochemical profile and its biological and therapeutic characteristics. Saffron is rich in carotenoids and terpenes. The major products of saffron are crocins and crocetin (carotenoids) deriving from zeaxanthin, pirocrocin and safranal, which give it its taste and aroma, respectively. Saffron and its major compounds have powerful antioxidant and anti-inflammatory properties in vitro and in vivo. Anti-tumor properties have also been described. The goal of this review is to present the beneficial effects of saffron and its main constituent molecules on neuropsychiatric diseases (depression, anxiety and schizophrenia) as well as on the most frequent age-related diseases (cardiovascular, ocular and neurodegenerative diseases, as well as sarcopenia). Overall, the phytochemical profile of saffron confers many beneficial virtues on human health and, in particular, on the prevention of age-related diseases, which is a major asset reinforcing the interest for this medicinal plant.

## 1. History and Geographical Distribution

*Crocus sativus* L. is a mythical aromatic medicinal plant whose stigmas provide saffron, also called ‘red gold’. A basic reference on the subject is a PhD thesis in Pharmacy by Claire Palomares [1]. The history of saffron goes back 3000–4000 years and is associated with several continents and civilizations. Saffron is found in Greco–Roman, Egyptian and Persian cultures. It is from Persia that saffron would have spread to India and China. It is only at the beginning of the middle ages, around the 9th century, with the Arab–Muslim conquests, that saffron spread in North Africa. With the invasion of Europe by the Moors, the cultivation of saffron spread in Europe and more particularly in Spain. The introduction of saffron in France is associated with the crusades between the 11th and 13th centuries. At that time, saffron was also cultivated in Germany, Switzerland and Italy. 

**Figure 1:**
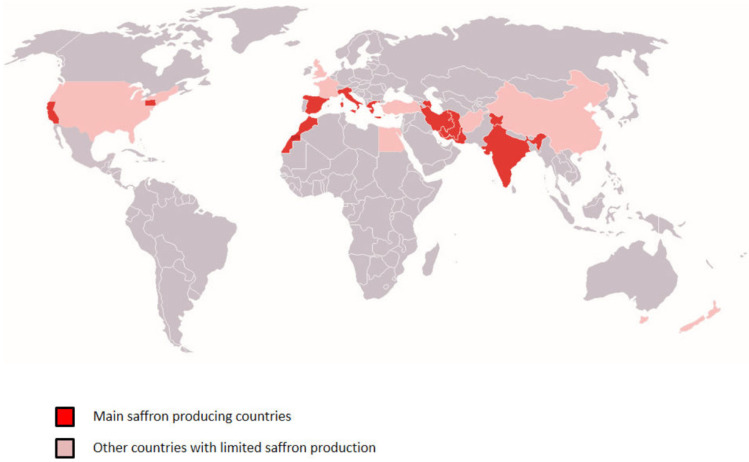
Main area of the culture of saffron in the world. (Realized by Nathalie Bancod (Université de Bourgogne, Dijon, France) based on the following site illustration: https://www.safranprestige.fr/histoire-du-safran/ (accessed on 6 January 2022)).

Currently, the main world producers of saffron are Iran, Greece, Morocco, Spain and India [1,2]. The largest global production is spread over a region from the Mediterranean basin to India (Figure 1). This geographical nuance was already mentioned in the Encyclopaedia of Diderot and d’Alembert [3]. To a much lesser extent, France, Switzerland, Italy, Turkey, Azerbaijan, Pakistan, China, Japan and the USA are included. 

## 2. Botany and Cultivation

Saffron is a plant widely described by botanists [4]. On several internet search engines, there is substantial agricultural and botanical information providing comprehensive information about saffron. Here, two examples from Morocco are provided [5,6]. The scientific name of saffron (*Crocus sativus* L.) was adopted by Linnaeus in 1754 [3]. Its taxonomy is as follows [1,7]:

* Kingdom: Plant;
* Phylum: Spermatophyte;
* Subphylum: Angiosperms (Magnoliophyta);
* Class: Monocotyledons (Liliopsida);
* Subclass: Liliidae; Order: Liliales;
* Family: Iridaceae;
* Subfamily: Crocoidae;
* Genus: Crocus (genus Crocus comprises about 90 species);
* Species: *C. Sativus* L.

**Figure 2:**
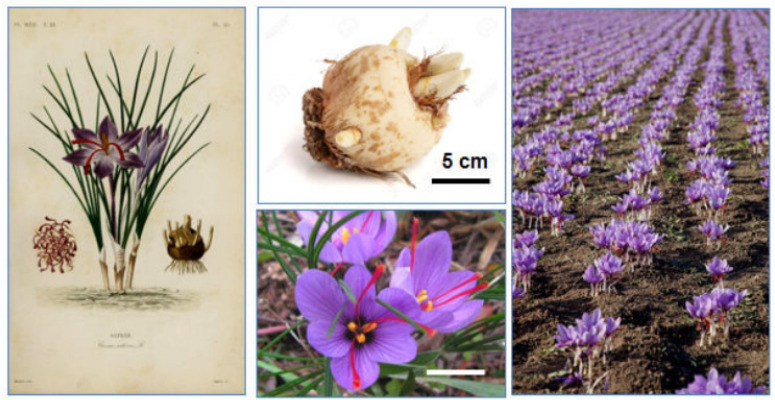
Aspects of saffron. Bulbous call cormus and flowers with stigmas are shown. The engraving corresponds to a picture of a botanical board (Illustration from Pierre-Oscar Reveil. Le règne végétal, Flore médicale, L. Guérin, Paris, 1864–1871).

Saffron is a plant illustrated on many botanical plates (Figure 2) and is a perennial and bulbous plant with a bulb called cormus. It can reach 30 cm in height, has long and thin leaves and its cup-shaped flowers are parma/purple in color (Figure 2). 

The reproduction of saffron is vegetative. Each cormus after flowering will degenerate and give birth in its upper part to several small corms. The saffron flowers in autumn with the number of flowers varying from 4–12 flowers/bulb. Each flower has three yellow pistils with three reddish-orange stigmas that give off a strong aromatic smell; these are the stigmas that provide the saffron. 

As saffron is a sterile plant, its perpetuation requires an intervention to support its multiplication. The cultivation of saffron is therefore almost totally dependent on man. It is a delicate culture, which requires a well-designed experiment and particular conditions. *Crocus sativus* L. appreciates the sunshine and fears the cold (lower than 15 °C). The soil must be calcareous or clayey with a pH between 6 and 7 [1,3,8]. The biggest enemies of saffron are certain animals (wild boars, rodents, etc.) and certain fungi. The maintenance of a saffron plantation requires vigilance and considerable effort. 

At present, new methods of cultivating saffron are being tested, which would make it possible to reduce its cost and decrease the fraud resulting from a high commercial demand. These new methods are also justified by climate change and the fight against pathogen aggression. Some producers have launched into hydropony or other methods of indoor cultivation (tissue culture) on artificial support other than soil. However, the consequences of these new methods of culture on the phytochemical characteristics of saffron are generally not well known, but the in vitro selection of plant producing elevated levels of bio-active compounds can open new perspectives in nutrition and for the development of new formulations containing the active substances of saffron (e.g., microencapsulation or nanoencapsulation). 

## 3. Harvesting Saffron

The flowers are picked by hand, which requires expert labor. After having been spread out for 0.5 to 1 day in a dry place, without excessive light and at a temperature of 20–30 °C, the stigmas of *Crocus sativus* L. are manually removed from the flower; this is the pruning stage. The stigmas are then dried (in the sun, oven, dessicator, etc.). This is a very delicate operation, which determines the quality of saffron. Saffron meets strict international quality standards: ISO 3632-1 and -2 2011. The quality of saffron is linked to its low water content and a high content of specific aroma (safranal) and specific coloring (crocin and picrocrocin) [3]. It is important to know that 1000 flowers of *Crocus sativus* L. will provide about 25 g of stigmas, which after drying will provide about 5 g of saffron [1,9]. 

## 4. Phytochemical Profile of Saffron 

The chemical characteristics of saffron, which has been used as a medicinal plant and in cooking for millennia [10], reveal the presence of more than 150 volatile and non-volatile elements. This includes carotenoids, a few polyphenols and flavonoids and terpenes whose proportions may vary depending on the country of origin [7,11,12,13,14,15]. The approximate composition is well summarized in previous studies [1,7,12]: water (10%); proteins and amino acids (12%); lipids (5%); minerals (5%); fibers (5%); various sugars (63%); and vitamins (B1 (riboflavin), B2 (thiamine)). The four major and biologically active compounds in saffron are crocin and crocetin (carotenoids deriving from zeaxanthin) [16,17], which give saffron its yellow color [18,19]; picrocrocin (apocarotenoid), which gives saffron its flavor [20]; and safranal (terpen with an aldehyde function) [14,21], which provides the specific odor of saffron. 

## 5. Physicochemical Properties of the Majority and Minority Compounds of Saffron

Thus, saffron is rich in carotenoids, which are powerful antioxidants capable, in particular, of neutralizing superoxide anions (O_2_**^•−^**) [22]. It also contains phenolic compounds capable of reacting with hydroxyl radicals (OH^•^) leading to their decrease [23] as well as flavonoids, which can inactivate superoxide, peroxyl (ROO^•^), alkoxyl (RO^•^) and hydroxyl radicals by hydrogen atom donation [24]. In addition, because of their capacity to chelate metal ions, such as iron and copper, flavonoids also inhibit free radical generation [25]. These antioxidant characteristics of the molecules present in saffron or saffron extracts have been demonstrated using different classical fluorometric and colorimetric tests, such as the oxygen radical absorbance capacity (ORAC) test, the ferric reducing antioxidant power (FRAP) test and the 2,2′-diphenyl-1-picrylhydrazyl (DPPH) test [26].

The synergistic effect of all the bioactive components present in saffron also exhibit significant antioxidant activities similar to those of vegetables rich in carotenoids. Our study provides an update on the antioxidant and anti-inflammatory properties of saffron related to its bioactive compounds, which can be useful to design different functional products in food, medicine and cosmetic industries [23].

## 6. Chemical Structures of the Main Compounds of Saffron

**Scheme 1:**
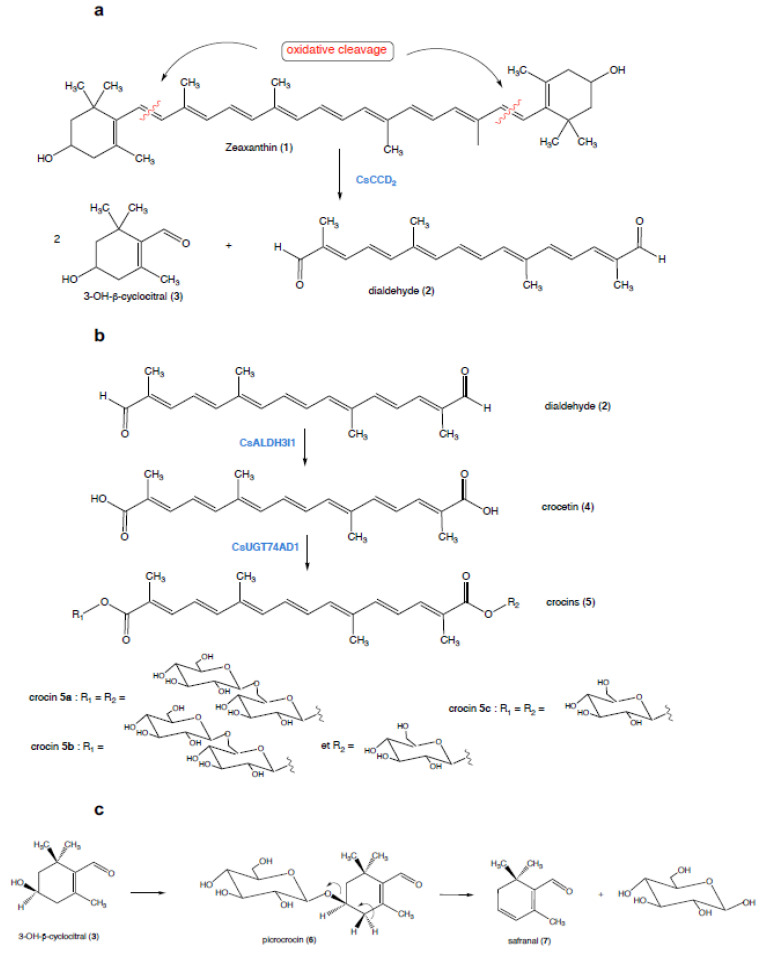
Biosynthesis of the main compounds (crocetin, crocins, picrocrocin, safranal) of saffron from zeaxanthin; (**a**) oxidative cleavage; (**b**) enzymatic modification in saffron; (**c**) different steps of safranal synthesis (Dominique Vervandier-Fasseur, Université de Bourgogne).

Zeaxanthin (**1**) is the “parent-molecule” of the four main compounds of saffron. Indeed, oxidative cleavage of (**1**) gives a carotenoid derivative, dialdehyde (**2**), and two apocarotenoid fragments, 3-OH-β-cyclocitral (**3**) (Scheme 1a). 

Oxidative transformation of (**2**) in diacid, named crocetin (**4**), followed with a di-esterification furnish crocins (**5**) (Scheme 1b). As shown in Scheme 1b, different molecules of crocin exist depending on the structure of the R1 and R2 radicals. The alcohol function of the apocarotenoid fragments (**3**) is transformed in glycosyl ether to give picrocrocin (**6**). Safranal (**7**) is obtained from (**6**) by the loss of a molecule of glucose (Scheme 1c). These transformations are carried out during the crocin biosynthesis and different enzymes are implied in the different steps [27]. However, the de-glycosylation of picrocrocin occurs mostly during the storage and air drying of fresh stigmas to give safranal [28].

Among the four main compounds of saffron, crocins (**5**) and picrocrocin (**6**) are bearing sugar moieties in the form of ester and ether functions, respectively. The presence of sugars in these chemical structures increases their water solubilities and makes their cell membrane crossing easier.

**Scheme 2:**
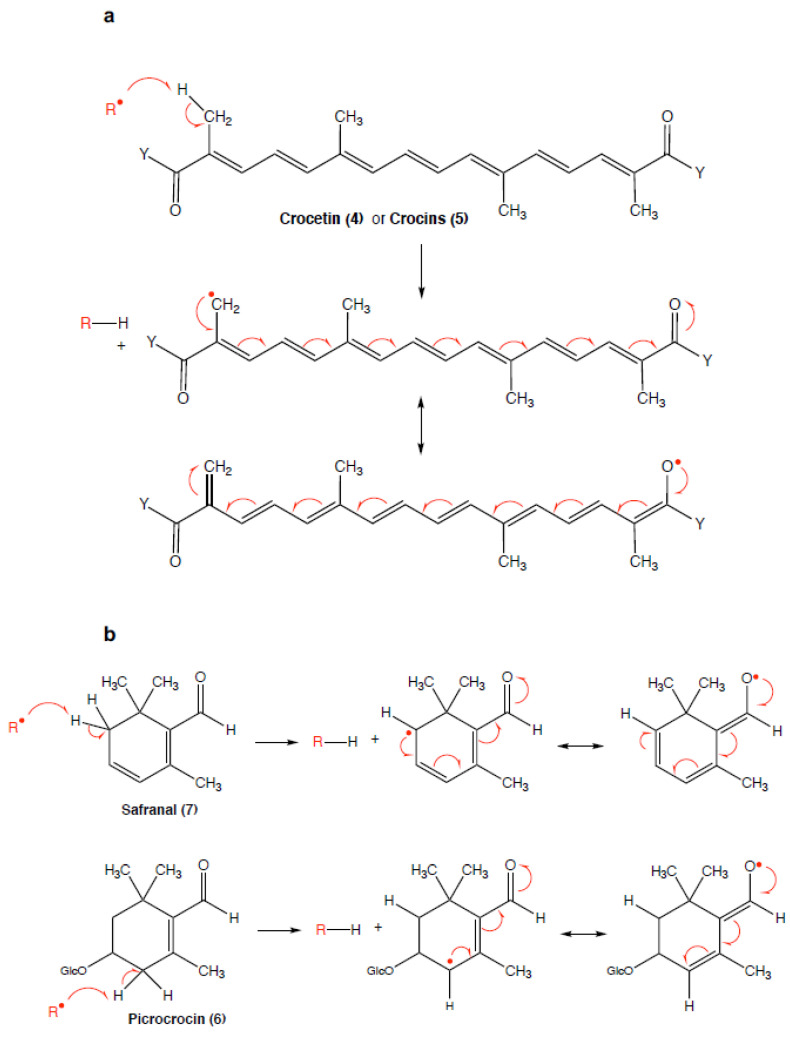
Hypothetical mechanism of radical formation and of its subsequent stabilization from the carotenoid and terpene chains; (**a**) radical attack on a carotenoid chain; (**b**) radical attack on a terpen structure (Dominique Vervandier-Fasseur, Université de Bourgogne).

Crocetin (**4**) and crocins (**5**) belong C-20 carotenoids. In contrast, safranal (**7**) provides a monocyclic terpene structure with an aldehyde function. As suggested in Scheme 2, both these chemical structures may easily give up a radical hydrogen H**^•^** to a free radical because the new radical is stabilized by the delocalization of the single electron on the carotenoid chain (Scheme 2a) or on the terpene chain (Scheme 2b). The different chemical features of the four main compounds of saffron helps to better understand their biological properties.

## 7. Biological Properties of Saffron Majority and Minority Compounds

### 7.1. Biological Properties of Crocetin and Crocins

Crocetin and crocins are important constituents of saffron. Crocins are members of a family of molecules which are formed from crocetin [27,29]. The safety of α-crocin (crocetin digentibiose ester, corresponding to crocin 5a on Scheme 1b), is well established. Thus, α-crocin has not shown toxic effects on hematological and biochemical parameters [30]. No mutagenic effects of α-crocin and dimethylcrocin have been observed in an Ames/Salmonella test carried out in a previous study [18]. In the literature, the type of crocin referred to is most often not precise or it corresponds to a mixture of crocins. Therefore, we refer to the general term ‘crocins’ in this paper since the information concerning the type of crocin used is not available.

Crocins are potent anti-inflammatory compounds [31]. On LPS-stimulated murine RAW264.7 macrophages, α-crocin inhibits cyclooxygenase-1 (COX1) and cyclooxygenase-2 (COX2), and blocks prostaglandine-2 (PGE2) production by inhibiting the nuclear translocation of NF-κΒ p50 and p65 sub-units [32]. On the same cell model, crocins also decrease inducible nitric synthase (iNOS) expression via Ca^2+^/calmodulin dependent protein kinase 4-PI3K/Akt-Nrf2 signaling cascade [33]. These anti-inflammatory properties have also been observed on different mice and rat models of asthma [34], neuroinflammation [35,36] and arthritis [37]. Crocins and crocetin are also known for their strong antioxidant activities [38]. Crocins and crocetin prevent the generation of free radicals, reduce lipid peroxidation and increase the levels of reduced glutathione and antioxidant enzymes (superoxide dismutase (SOD), catalase (CAT) and glutathione peroxidase (GPx)). On acrylamide-treated PC12 cells, crocins prevent reactive oxygen species’ (ROS) overproduction and apoptosis [39]. Crocins also prevent ROS overproduction on PC12 cells cultured in the presence of high glucose concentrations [40]. In addition, on PC12 cells deprived of serum and glucose, crocins inhibit lipid peroxidation and partly restore SOD activity at the same concentration and were more efficient than α-tocopherol [41].

**Figure 3:**
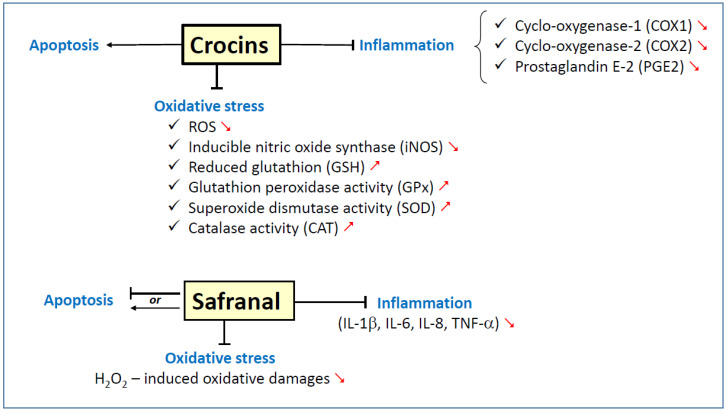
Beneficial effects of crocin and safranal molecules on inflammation, oxidative stress and apoptosis. In different cellular and animal models, crocin molecules counteract inflammation by reducing the production of COX1 and 2 as well as PGE2, oppose oxidative stress by decreasing the production of ROS and iNOS and by stimulating antioxidant defenses (increase in GSH, GPx, SOD and CAT levels). As for safranal, it reduces the production of pro-inflammatory cytokines and reduces oxidative stress stimulated by H_2_O_2_. Moreover, crocins induce apoptosis, while safranal activates or inhibits cell death, depending on the models used. The signaling pathways on which these molecules act are still not well known but the PI3K/Akt and MAPK/ERK pathways could be involved. ↗ means increase, ↘ means decrease.

It is notable that pharmacokinetic studies have shown that crocins are not available after oral administration in blood circulation. Indeed, crocins are converted into crocetin in the intestine but the level of crocetin in the plasma is low. However, crocetin can distribute in different tissues because of its weak interaction with albumin. Crocetin can cross the blood–brain barrier and reach the central nervous system by passive transcellular diffusion and can therefore be effective in neurodegenerative disorders. A large amount of crocins is eliminated in the feces [12]. To improve crocin and crocetin stability as well as bioavailability, various nanotechnological approaches are currently being evaluated [42]. The main biological properties of crocins are summarized in Figure 3. 

### 7.2. Biological Properties of Picrocrocin and Safranal

Chemical structures of picrocrocin and crocins were compared in a study on neuronal injury in vitro and in vivo [43]. Both compounds of saffron bear glucosyl and/or gentiobiosyl fragments, which allow for the increase in water solubility and enhance cell membrane crossing. However, cleavage of ester functions (case of crocins) are easier than cleavage of ether functions (case of picrocrocin). Thus, liberation in cell medium of crocetin, a carotenoid compound known for its antioxidant activities, may explain the fact that antioxidant properties of saffron are mainly due to crocins and not picrocrocin. In human monocytic U397 cells, picrocrocin was not found to act against oxidant-induced DNA damage [43]. In contrast, picrocrocin reduced the proliferation of Caco-2 and HepG2 cells [44]. Apoptosis of HepG2 cells may be caused by safranal by an endoplasmic reticulum stress and this same compound may promote DNA damage in inducing a double DNA strand break [45]. 

The terpenoid structure of safranal provides antioxidant properties. Some studies highlight the antioxidant activities of safranal and some others the opposite. Thereby, the effect of saffron on H_2_O_2_-induced toxicity in human neuroblastoma SH-SY5Y cells is only due to antioxidant activity of crocetin; indeed, safranal was shown to play no role in repressing ROS production and decreasing caspase-3 activation [46]. In contrast, in the case of type-2 diabetes and diabetic nephropathy, safranal was found to exert both antioxidant and anti-inflammatory effects in renal tissue. In an experiment on type-2 diabetes in rats induced by streptozotocin, safranal was shown to be an antioxidant and an anti-inflammatory agent: safranal treatment decreased the nitric oxide (NO) level as well as the TNF-α and IL-1β levels [47]. The inflammatory markers, including IL-1β, IL-6, IL-8, TNF-α and NF-κΒ are features of neurodegenerative diseases. A study of beneficial effects on cognitive deficits after an intra-hippocampal injection of beta-amyloid (Aβ_1-40_) in rats highlighted the decrease of their levels after safranal treatment [48]. Hippocampal Aβ_1-40_ injury may increase levels of deleterious free radicals, which lead to mitochondrial dysfunction. Baluchnejadmojarad et al. determined the mitochondrial membrane potential (MMP) after safranal treatment and showed that this active saffron extract may prevent MMP decrease and therefore, protect mitochondrial activity [48]. PC12 cells can be used as in vitro model of Alzheimer’s disease. When PC12 cells are exposed to beta-amyloid, safranal treatment decreased cell apoptosis induced by beta-amyloid through P13K/AKT and MAPK/ERK pathways [49]. In this same study, the authors showed the protective effect of safranal against free radicals produced by H_2_O_2_, in PC12 cells. These results bear out the results of Hosseinzadeh et al. about the protective role of safranal on oxidative damage in hippocampal tissue of rats due to cerebral ischemic insult [50]. Ca^2+^ excess can be involved in the myocardial ischemia by increasing the contractility in the myocardium. In addition, in mitochondria, Ca^2+^ excess can lead to overproduction of ROS. The relaxant activity of safranal was highlighted in isolated rat aorta. Inhibition of the smooth muscle cell contraction was due to the blockage of the L-type calcium channels (LTCC) by an endothelium-independent mechanism [51]. These results were confirmed by an in vivo study about the role of safranal in cardiovascular protection: in reducing activity of LTCC in cardiomyocytes membranes, safranal was considered as a Ca^2+^ channel antagonist. Thus, these data support that this main compound of saffron can regulate Ca^2+^ homeostasis and oxidative stress [52]. The main biological activities of safranal are summarized in Figure 3. 

## 8. Benefits of Saffron on Human Health

**Figure 4:**
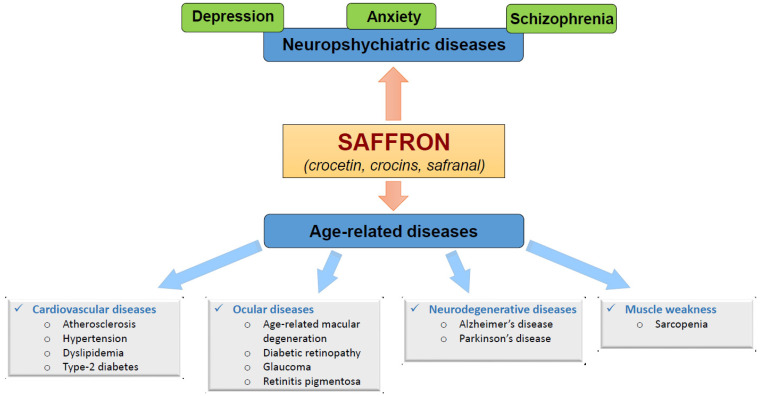
Beneficial effects of saffron constituents (crocetin, crocins and safranal) on neuropsychiatric and age-related diseases.

Avicenna (famous Persian physician of the 10th century) described various uses of saffron, including its use on inflammatory and respiratory diseases as well as its benefits on sexual activities (aphrodisiac properties); most of these effects have been studied in modern pharmacology and are well documented [53,54]. Currently, the impact of saffron on the central nervous system, mainly on mental diseases, is widely studied and numerous data are available [55]. There is also substantial evidence showing that saffron has several benefits on age-related diseases, including cardiovascular diseases [56,57], ocular diseases [58,59], neurodegenerative diseases [60,61] and type-2 diabetes [62]. The main beneficial effects of major saffron constituents (crocetin, crocins and safranal) on neuropsychiatric and age-related diseases are summarized in Figure 4. 

### 8.1. Benefits of Saffron on Neuropsychiatric-Diseases

Several studies have examined the effects of saffron on neuropsychiatric diseases. These investigations have suggested that saffron constitutes an effective treatment for depression, anxiety and schizophrenia [63,64,65,66,67,68].

#### 8.1.1. Depression

Studies have reported that saffron extracts and their constituents, safranal and crocins, exert antidepressant effects through an activation of serotonergic, noradrenergic and dopaminergic systems in mice submitted to the forced swim test [69]. Crocetin was found to be efficient in the treatment of depression in mice [70]. In addition, a randomized controlled trial showed that 50 mg saffron resulted in a significant decrease in the Beck depression and anxiety inventory scores in comparison to placebo treatment [62]. Moreover, combined treatment with saffron and curcumin was found to be associated with greater improvements in depressive symptoms in patients suffering from major depression disorder compared to a placebo [71].

#### 8.1.2. Anxiety

Investigations have shown that aqueous saffron extracts and its constituent safranal exert anxiolytic effects similar to that of diazepam, probably through their interaction with the benzodiazepine binding site at the GABA_A_ receptor [72,73,74]. In addition, studies have demonstrated that crocins alleviated the obsessive compulsive behavior in rats through an antagonistic action at the 5-HT2C receptor site [75].

#### 8.1.3. Schizophrenia

Animal studies have reported that acute supplementation with crocins improved the schizophrenia negative symptoms as reflected by the attenuation in the social isolation induced by sub-chronic treatment with ketamine [67]. Moreover, crocins were found to attenuate the psychotomimetic effects (hypermotility, stereotypies and ataxia) as well as the recognition memory deficits induced by ketamine in rats [67]. In the frame of one double-blind, placebo-controlled study, saffron aqueous extract and crocins treatments for 12 weeks were found to be safe and well tolerated in schizophrenic patients [68].

## 9. Benefits of Saffron on the Prevention of Age-Related Diseases 

### 9.1. Benefits of Saffron on Cardiovascular Diseases

The analysis of publications from different databases shows that saffron has antioxidant, anti-inflammatory, anti-hypertensive and hypolipidemic properties [56,76,77]. These different properties are in favor of beneficial effects of saffron to prevent, or even treat, cardiovascular diseases.

Animal studies have reported that crocetin exerts protective effects against myocardial ischemia reperfusion injury by inhibiting malondialdehyde (MDA) production, blocking tumor necrosis factor-alpha (TNF-α) activity and reducing myocardium apoptosis and infarct size [78]. Investigations have shown that saffron and safranal exert cardioprotective effects in isoproterenol-induced myocardial infarction through the modulation of oxidative stress in Wistar rats [79]. Moreover, saffron was found to attenuate the susceptibility and incidence of fatal ventricular arrhythmia in ischemia-reperfusion rat models [80]. In addition, saffron was found to induce neuroprotective effects on late cerebral ischemia in association with the attenuation in astrogliosis and glial scar formation in ischemic stroke rat models [81]. Higashino et al. [82] reported that crocetin exerted antithrombotic effects through an increase in nitric oxide (NO) bioavailability in stroke-prone spontaneously hypertensive rats. Moreover, studies have shown that safranal and crocins decreased the infarct volume in the transient cerebral ischemia rat model mainly by suppressing the production of free radicals and increasing antioxidant activity [83,84]. Interestingly, two recent randomized clinical trials showed that acute and chronic treatment of saffron aqueous extracts, combined with stroke care, reduced the NIHSS (National Institutes of Health Stroke Scale) in association with decreased MDA or brain-derived neurotrophic factor (BDNF) serum levels in ischemic stroke patients [85,86]. In relation to cardiovascular diseases, various benefits of saffron have been described for atherosclerosis, hypertension, dyslipidemia and type-2 diabetes, which are major risk factors in the development of cardiovascular diseases.

#### 9.1.1. Atherosclerosis

Atherosclerosis is a major histological modification of the arteries, which favors cardiovascular diseases and vascular dementia [87,88]. The identification of drugs allowing for the prevention of atherosclerosis is therefore of major interest. Crocetin was found to decrease the low density lipoprotein (LDL) oxidation and to reduce the vascular thiobarbituric reactive acid substances (TBARs), NF-κΒ activation, vascular cell adhesion molecule-1 (VCAM-1) expression, foam cell formation and the progression of aortic atherosclerotic lesions in rabbits treated with a high fat diet for 8 weeks [89]. The anti-atherosclerotic effect of crocetin was explained in part by the inhibition of NF-κΒ activation and VCAM-1 expression, a cytokine-inducible member of immunoglobulin gene superfamily, implicated in atherogenesis by favoring adhesion of monocytes to vascular endothelium, subsequently facilitating the extravasation into the subendothelial space [89,90]. Moreover, saffron was found to prevent atherosclerosis in high fat treated rats through the suppression of p38 mitogen-activated protein kinase (MAPK) pathway and inhibition of smooth muscle cell proliferation [91]. It is well known that endothelial dysfunction, which is characterized by a reduction in the bioavailability of nitric oxide (NO), is an early step in the development of atherosclerosis [92]. Tang et al. [93] have suggested that crocetin may exert its beneficial effects on atherosclerosis through an upregulation of endothelial nitric oxide synthase (eNOS) expression, thus alleviating the development of LDL oxidation-induced endothelial dysfunction. Moreover, recent studies have shown that saffron aqueous extract attenuated the progression of aortic stenosis and improved the plaque stability in apoE knockout (ApoE^−/−^) atherosclerotic mice [94]. The anti-atherosclerotic effects were explained partly by the reduction in IL-6, tumor necrosis factor-α (TNF-α) and monocyte chemoattractant protein-1 (MCP-1) expressions most likely leading to decreased formation of foam cells [94]. In the frame of a randomized placebo-controlled clinical trial, crocins treatment (30 mg/day) for 8 weeks was found to decrease the oxidized-LDL and MCP-1 levels in patients with coronary artery disease [95].

#### 9.1.2. Hypertension

Hypertension often associated with cardiovascular diseases can be associated with atherosclerosis in certain patients. Previous studies have reported that saffron extract and its components, notably safranal and crocins, reduced the mean arterial blood pressure in desoxycorticosterone acetate (DOCA), salt induced hypertensive rats [96], in a dose-dependent manner. The same authors have shown that chronic treatment with safranal decreased the systolic blood pressure in desoxycorticosterone acetate (DOCA), salt induced hypertensive rats [97]. Moreover, investigators have suggested that saffron induces relaxation in isolated aortic rings through mainly its effect on endothelium via nitric oxide synthase pathway [98]. Recent studies have demonstrated that saffron reduces the blood pressure in an angiotensin II-induced hypertension rat model through an inhibition of the renin–angiotensin system [99]. Crocetin treatment for 3 weeks was found to decrease the systolic blood pressure in spontaneously hypertensive rats [82]. The antihypertensive effect of crocetin was suggested to be related to an increase in NO bioavailability [82]. In addition, in the frame of a double-blind, placebo-controlled study, saffron (400 mg) treatment for seven days decreased the systolic blood pressure and the mean arterial pressure in healthy humans [100]. More recently, saffron (200 mg per day) supplementation for 12 weeks was found to improve blood pressure in elderly hypertensive subjects [101]. The underlying mechanism for which saffron exerts its beneficial effect on blood pressure may be explained in part by the decrease in endothelin-1 (ET-1) levels observed in elderly patients treated with saffron [101].

#### 9.1.3. Dyslipidemia

Dyslipidemia associated with enhanced LDL cholesterol and/or triglyceride levels is a major risk factor in cardiovascular diseases and strongly contributes to the different steps of atherosclerosis, from its initiation (fatty streak) to its final step (atheromatous plaque). Studies have shown that saffron and crocin decrease the elevated levels of triglycerides (TG), total cholesterol (TC) and LDL cholesterol in high fat treated rats [102]. The mechanism underlying these effects was attributed to the modulation of oxidative stress as reflected by the reduction in the rise of MDA and an increase in antioxidant enzymes in high fat fed rats treated either with saffron or crocins [102]. Moreover, 10-day treatment with crocins was found to reduce serum TG, TC, LDL cholesterol and very low density lipoprotein (VLDL) cholesterol levels in high fat-induced hyperlipidemic rats. The authors of this study suggest that crocins exert their hypolipidemic effects partly through the mal-absorption of fat and cholesterol induced by an inhibition of pancreatic lipase [103]. In addition, one recent meta-analysis of randomized controlled trials has shown that supplementation with saffron resulted in a reduction in serum concentrations of TG and TC with an increase in HDL cholesterol [104].

#### 9.1.4. Type-2 Diabetes

Type-2 diabetes is not only a risk factor for cardiovascular diseases but also for dementia. Studies undertaken on animals have shown that saffron improves the insulin levels and lipid profile in obese insulin resistant rats. The beneficial effects of saffron on insulin resistance appeared to be associated to a decrease in oxidative stress and normalization in adiponectin levels [105]. Crocetin was also found to prevent the development of insulin resistance in fructose fed rats [106]. The beneficial effects of crocetin on insulin sensitivity appeared to be associated to the normalization of the decreased protein and mRNA expression of adiponectin at the level of adipose tissue in fructose fed rats [106]. In addition, in the frame of a randomized double-blind clinical trial, saffron (15 mg per day) supplementation for 3 months was found to significantly decrease the fasting plasma glucose, hemoglobin A1c (HbA1c), total cholesterol, LDL-cholesterol and LDL/HDL ratio in type-2 diabetic patients in comparison to baseline group [107]. The discrepancy to find similar results in the effects of saffron on glucose control parameters in other clinical studies could be explained by using different saffron concentrations, treatment durations and population samples [108,109,110].

### 9.2. Benefits of Saffron on Ocular Diseases

Several studies have underlined the potential beneficial effects of saffron in various models of ocular diseases, such as age-related macular degeneration (AMD), diabetic retinopathy, and glaucoma, which are frequent age-related diseases [111,112] as well as in retinitis pigmentosa, which is a genetic disorder of the eye [113,114,115,116].

#### 9.2.1. Age-Related Macular Degeneration

Among the ocular degenerative diseases, AMD is currently considered as the leading cause of irreversible vision loss in developed countries [117]. Studies have shown that saffron is effective in reducing retinal degeneration in bright continuous light exposure rat models, an AMD experimental model [118]. This neuroprotective effect of saffron was thought to be attributed to its antioxidant properties and its involvement in the regulation of genes, which control the release of pro-inflammatory cytokines by glial cells [119]. Saffron treatment (20 mg/day) was found to improve focal electroretinogram (fERG) findings in one placebo-controlled study of patients with AMD [120]. Furthermore, 14 months follow-up of these patients demonstrated ongoing saffron treatment ameliorated visual acuity and fERG parameters [121]. However, in the frame of a randomized, double-blind, placebo-controlled crossover trial, saffron supplementation modestly improved the visual function in patients suffering from AMD [114]. The failure to significantly delay the progression of such chronic disease probably requires a longer period of treatment with saffron.

#### 9.2.2. Diabetic Retinopathy

In streptozotocin treated rats, a model of diabetic retinopathy, saffron extract was found to protect the antioxidant reserve and to decrease lipid peroxidation in retina tissue [122]. Moreover, studies have shown that crocins decrease microglial activation in high glucose-free fatty acid BV-2 and N9 cultured cells through its antioxidative and anti-inflammatory properties [123]. The authors suggest that crocins could be used as a promising medicine agent in controlling microglial activation in diabetic retinopathy [123]. 

#### 9.2.3. Glaucoma

Studies have shown that oral saffron extract administration was able to reduce the rise in intraocular pressure and to prevent the retinal ganglion cell death in a model of experimental glaucoma [124]. It has been postulated that this neuroprotective effect of the saffron extract could be due to its anti-inflammatory effects and its antioxidant properties [124]. In the frame of a randomized interventional pilot study, oral aqueous saffron extract was found to exert an ocular hypotensive effect in patients suffering from primary open-angle glaucoma [116]. Further studies should be undertaken to determine the mechanisms for which topical saffron components could ameliorate the glaucoma disease.

#### 9.2.4. Retinitis Pigmentosa

Retinitis pigmentosa is a genetic degenerative disease. Investigations have demonstrated that dietary supplementation with safranal slows photoreceptor cell degeneration and improves the loss of retinal function and vascular network disruption in the P23H rat models of autosomal dominant retinitis pigmentosa [125]. The underlying mechanisms for which saffron exerts its beneficial effects appears to be mediated through the prevention of the increase in peroxide-induced oxidative damage and inhibition of caspase-mediated apoptosis [126,127].

### 9.3. Benefits of Saffron on Neurodegenerative Diseases

There are also several arguments supporting the benefits of saffron on the treatment of two major age-related neurodegenerative diseases, Alzheimer’s disease and Parkinson’s disease, either in human or in animal models.

#### 9.3.1. Alzheimer’s Disease

Saffron and crocins were found to inhibit beta-amyloid aggregation, a key step in the pathogenesis of Alzheimer’s disease [128,129]. In an Alzheimer’s disease rat model induced by streptozocin intra-cerebroventricularly, studies have shown that crocins (30 mg/kg) are effective in antagonizing the learning and memory impairments. Moreover, recent investigations have shown crocins to improve cognition and memory abilities and reduce Aβ_1-42_ content in cerebral cortex and hippocampus in a mouse model of Alzheimer’s disease induced by D-galactose and aluminum trichloride [130]. The same study showed crocins to increase the levels of glutathione peroxidase, superoxide dismutase and choline acetyltransferase, and decrease the levels of ROS and acetylcholinesterase in the cerebral cortex and the hypothalamus. The authors concluded that crocins seem to exert beneficial effects on the development of Alzheimer’s disease partly through its antioxidant and anti-apoptotic properties [130]. In the frame of a double-blind-placebo-controlled study, 30 mg per day supplementation with saffron for 16 weeks resulted in improved cognitive function in patients suffering from mild to moderate Alzheimer’s disease [131]. Moreover, the follow-up of this study in which the authors evaluated the effects of saffron (30 mg/day) for 22 weeks showed that saffron was as effective as donepezil in the treatment of mild-to-moderate Alzheimer’s disease [132]. It is noteworthy that saffron extract and donepezil treated patients presented similar adverse events (AEs) frequency with the exception of vomiting, which occurred more frequently in the donepezil group [129]. In addition, a recent systematic review of clinical trials demonstrated that saffron was equally effective as commonly used drugs for Alzheimer’s disease and resulted in no difference in the incidence of side effects [133].

#### 9.3.2. Parkinson’s Disease

The characterized neuropathology of Parkinson’s disease patients is the selective progressive degeneration of dopaminergic neurons in the substantia nigra (SN) leading to a depletion of projecting dopaminergic nerve fibers in the striatum [134]. Moreover, accumulation of endogenous 6-hydroxydopamine (6-OHDA), which is a neurotoxin that selectively destroys dopaminergic and noradrenergic neurons in the brain, has been identified in patients with Parkinson’s disease [135]. Interestingly, studies have reported that crocetin protected the substantia nigra neurons against the deleterious effects of 6-OHDA through the preservation of reduced glutathione (GSH) and dopamine levels and the attenuation of TBARs in a 6-hydroxydopamine (6-OHDA) rat model Parkinson’s disease [136]. Moreover, in the same animal model, crocins improve memory performance. The authors suggested that the beneficial effects exerted by crocins on memory in Parkinsonian rats, are mediated, at least in part, through reducing TBARs and nitrite (NO_2_^−^) levels in the hippocampus [137]. In another animal model of Parkinson’s disease induced by 1-methyl-4-phenyl-1,2,3,6-tetrahydropyridine (MPTP), crocetin treatment was found to attenuate the motor deficits and to preserve dopaminergic neurons in association with the protection against mitochondrial dysfunction by blocking the mitochondrial permeability transition pore (mPTP) opening [138].

#### 9.3.3. Prevention of Muscle Weakness in the Elderly

Muscle weakness is generally defined as a temporary or permanent loss of muscle strength. Under its more severe form, it is defined as sarcopenia [139]. It is usually due to a lack of exercise, muscle injury, pregnancy or aging. It can also occur with long-term conditions, such as diabetes or heart disease. The effect of saffron in the management of muscle weakness has been described. In this context, the effect of saffron on physical performance in 28 healthy men has been studied. This study indicated that saffron supplementation (300 mg/day or 10 days) increased muscle strength and improved reaction time. Results indicated that saffron treatment improved mitochondrial function as well as blood flow and oxygen delivery to the muscles during exercise [140]. Previous studies have suggested that saffron contains two beneficial carotenoids called crocin(s) and crocetin [141], which are associated with the prevention of muscle fatigue and weakness. On this basis, Lei et al. indicated that crocins relieve pain and muscle suffering in osteoarthritis rats triggered by MNX surgery [142]. They also showed that crocins can reduce oxidative stress and inflammation induced by osteoarthritis. Oxidative stress may be involved in the pathogenesis of muscle dysfunction and inflammation. Indeed, ROS oxidize various components of the cell and can lead to cellular injury and even cell death [143]. The result of these disturbances is a cellular dysfunction leading to inflammatory disorders [144]. The elevation of ROS production and alteration of antioxidant enzyme systems leads to muscle loss and weakness [145]. Crocins treatment attenuated oxidative stress and improved the muscle strength. It has been reported that crocins can increase the activities of GSH reductase and gamma-glutamyl cysteine synthetase (gamma-GCS); hence, it can contribute to a stable GSH [43,142]. Moreover, it has been shown that crocins have an important relaxing effect on rat tracheal smooth muscle cells [146]. Crocetin was also found to effectively treat and prevent physical fatigue in men [147]. Previous studies have shown that crocetin is a potent antioxidant [148]. Indeed, ROS are responsible for exercise-induced protein oxidation and contribute to physical fatigue. Therefore, supplementation of crocetin can alleviate physical fatigue through its antioxidant function [147].

## 10. Conclusions

Saffron’s rich phytochemical profile provides a promising approach in the prevention and treatment of age-related diseases, thus reinforcing the interest for this medicinal plant. Indeed, saffron and especially its main constituent molecules (crocins, crocetin, picrocrocin and safranal) exert beneficial effects on frequent neuropsychiatric (depression, anxiety, schizophrenia, etc.) and age-related (cardiovascular, ocular, neurodegenerative diseases and sarcopenia) diseases. In a nutritional and therapeutic context, the clarification of the molecular mechanisms by which saffron and its compounds exert their beneficial effects will make it possible to optimize their effectiveness and rationalize their use for the benefit of human health.
